# Robotic versus laparoscopic surgery for colorectal disease: a systematic review, meta-analysis and meta-regression of randomised controlled trials

**DOI:** 10.1308/rcsann.2024.0038

**Published:** 2024-05-24

**Authors:** A Thrikandiyur, G Kourounis, S Tingle, P Thambi

**Affiliations:** ^1^ South Tees Hospitals NHS Foundation Trust, UK; ^2^ Newcastle University, UK

**Keywords:** robotic surgery, laparoscopic surgery, colorectal disease, postoperative outcomes, complications

## Abstract

**Introduction:**

Robotic surgery (RS) is gaining prominence in colorectal procedures owing to advantages like three-dimensional vision and enhanced dexterity, particularly in rectal surgery. Although recent reviews report similar outcomes between laparoscopic surgery (LS) and RS, this study investigates the evolving trends in outcomes over time, paralleling the increasing experience in RS.

**Methods:**

A systematic review, meta-analysis and meta-regression were conducted of randomised controlled trials exploring postoperative outcomes in patients undergoing RS or LS for colorectal pathology. The primary outcome measure was postoperative complications. Risk of bias was evaluated using the Cochrane Collaboration’s assessment tool. Randomised controlled trials were identified from the PubMed^®^, Embase^®^ and CINAHL^®^ (Cumulative Index to Nursing and Allied Health Literature) databases via the Cochrane Central Register of Controlled Trials.

**Results:**

Of 491 articles screened, 13 fulfilled the inclusion criteria. Meta-analysis of postoperative complications revealed no significant difference between RS and LS (relative risk [RR]: 0.96, 95% confidence interval [CI]: 0.79 to 1.18, *p*=0.72). Meta-regression analysis of postoperative complications demonstrated a significant trend favouring RS over time (yearly change in Ln(RR): −0.0620, 95% CI: −0.1057 to −0.0183, *p*=0.005). Secondary outcome measures included operative time, length of stay, blood loss, conversion to open surgery, positive circumferential resection margins and lymph nodes retrieved. The only significant findings were shorter operative time favouring LS (mean difference: 41.48 minutes, 95% CI: 22.15 to 60.81 minutes, *p*<0.001) and fewer conversions favouring RS (RR: 0.57, 95% CI: 0.37 to 0.85, *p*=0.007).

**Conclusions:**

As experience in RS grows, evidence suggests an increasing safety profile for patients. Meta-regression revealed a significant temporal trend with complication rates favouring RS over LS. Heterogeneous reporting of complications hindered subgroup analysis of minor and major complications. LS remains quicker. Rising adoption of RS coupled with emerging evidence is expected to further elucidate its clinical efficacy.

## Introduction

Colorectal disease management has long relied on surgical interventions as the cornerstone of curative treatment. The advent of minimally invasive surgery has rapidly gained prominence, demonstrating improved clinical outcomes compared with traditional open procedures.^1^ In particular, studies from individual institutions suggest that laparoscopic and robotic colectomies yield equivalent (and in some cases superior) clinical results.^2,3^

However, conventional laparoscopic surgery is not without limitations, marked by two-dimensional operating views, reduced dexterity, unstable video camera imaging and a long initial training period. Addressing these challenges, robotic surgery has emerged with distinctive advantages, including three-dimensional imaging, ambidexterity, enhanced robotic arm manoeuvrability and a shorter learning curve.^4,5^ Consequently, robotic surgery has witnessed increased adoption, especially in colorectal procedures involving patients with rectal pathology.^6^

While numerous studies have compared robotic and laparoscopic techniques for colorectal surgery,^7,8^ a meta-analysis by Tang *et al* in 2021 concluded that both modalities exhibit comparable outcomes, notably in terms of postoperative complications within 30 days.^9^ As robotic surgeons gain proficiency and independence due to the recognised shorter learning curve associated with robotic surgery, the existing literature lacks investigations into the temporal trends in complication rates. In order to address this gap, we conducted a meta-analysis supplemented with a meta-regression analysis, focusing on postoperative complications as the primary outcome measure. This approach allows us to explore the changing landscape of outcomes over time and provides valuable insights into the trajectory of complication rates associated with robotic colorectal surgery.

## Methods

The study was conducted in accordance with the 2020 PRISMA (Preferred Reporting Items for Systematic reviews and Meta-Analysis) statement.^10^ The research question was formulated using the PICO (Population, Intervention, Comparison, Outcome) approach. The protocol was registered on the PROSPERO database (CRD42023447954). Ethical approval was not required.

### Identification and selection of studies

The PubMed^®^, Embase^®^ and CINAHL^®^ (Cumulative Index to Nursing and Allied Health Literature) databases were searched via the Cochrane Central Register of Controlled Trials. The search terms were “robo* AND (colorectal OR gastr*)” and the databases were queried from inception up to 25 July 2023. Two independent authors (GK and AT) screened the articles generated from the initial search. Duplicate and other irrelevant studies were removed. The two authors further reviewed the eligible studies either in full paper form or as abstracts. Disagreements regarding the studies were resolved by discussion.

Papers written in languages other than English were included if they had a corresponding abstract in English. Review articles were not included but their references were used to find studies that met our inclusion criteria. The studies that were finally included in the review were those that were randomised controlled trials (RCTs), had a direct comparison of laparoscopic and robotic operations in colorectal surgery, and had clearly reported postoperative outcomes including the primary outcome of postoperative complications. The full inclusion and exclusion criteria are set out in Table 1.

**Table 1:** Inclusion and exclusion criteria for review

	Inclusion criteria	Exclusion criteria
Population	Adults (age ≥16 years) with colorectal pathology requiring surgical intervention	Age <16 years
Intervention	Robotic colorectal surgery (malignant or benign)	Minimally invasive surgery not including colon or rectum
Control	Laparoscopic surgery	Open surgery
Outcome	Primary outcome: postoperative complicationsSecondary outcomes: operative time, blood loss, lymph nodes retrieved, positive circumferential resection margin, conversion to open surgery, postoperative pain, length of stay	Primary outcome not reported
Study design	Randomised controlled trials	Cohort studies, non-randomised trials, propensity-matched studies, review articles
Other	Any language as long as there was an English abstract	

### Data extraction

The following data were extracted independently by two authors (GK and AT) and then reviewed:

– Individual study particulars – study authors, publication year, country of study, sample size, colorectal pathology being treated
– Primary outcome – overall postoperative complications within the short-term perioperative period
– Secondary outcomes – operative time, blood loss, lymph nodes retrieved, circumferential resection margin positive status, conversion to open surgery, postoperative pain and length of hospital stay

Discrepancies between studies were discussed and appropriate studies were further excluded.

### Statistical analysis

The quantitative analysis and synthesis of the included studies was conducted using R statistical software (R Foundation for Statistical Computing, Vienna, Austria; www.r-project.org) with the tidyverse^11^ and metafor^12^ packages, along with RevMan 5.4.1 (Nordic Cochrane Centre, Copenhagen, Denmark). A *p*-value of <0.05 was deemed statistically significant. In order to assess heterogeneity among studies, the I^2^ statistic was employed, with I^2^>50% defined as significant heterogeneity. Where means and standard deviations were not reported in the manuscript, these were calculated using the reported medians, ranges and interquartile ranges, and the Cochrane calculators in RevMan 5.4.1.

The Mantel–Haenszel random-effects model was employed for meta-analyses of dichotomous outcomes, such as complication rates, and results were presented as relative risks (RR) with 95% confidence intervals (CI). For continuous scale outcomes, such as operative time, inverse variance random-effects models were applied, and results were reported as mean differences (MD) with 95% CI. In cases of outcomes reported on different scales, standard mean differences (SMD) with 95% CI were provided. Meta-regression was used to assess whether any differences between robotic and laparoscopic approaches were modified by year of study publication (i.e. to identify any temporal trends). For meta-regression analysis, the mixed-effects model (using the regplot() function of the metafor package) was employed to fit the postoperative outcomes, with publication year as the moderator. RR for binary outcomes was transformed using a natural logarithmic transformation for the meta-regression analysis.

### Risk of bias assessment

Two independent authors (GK and AT) conducted a review of the included RCTs and evaluated their risk of bias using the Cochrane Collaboration’s assessment tool.^13^ The domains assessed included selection bias, performance bias, detection bias, attrition bias, reporting bias and other bias. Using the Cochrane tool, trials were categorised within each domain as having a high, low or unclear risk of bias, with the latter occurring in instances where inadequate information was given in the trials to make a judgement.

## Results

**Figure 1:**
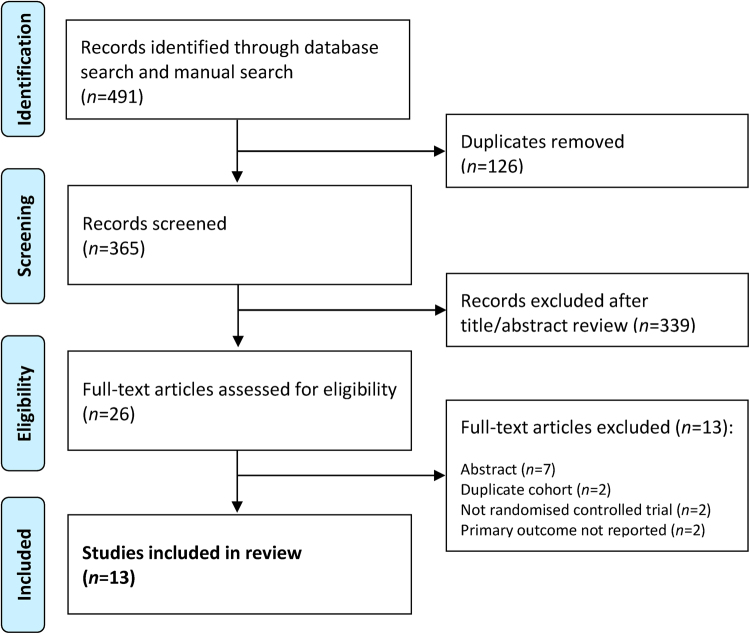
Flowchart of study selection

A total of 491 records were identified in the initial search. On eliminating duplicate entries, the abstracts of 365 papers were screened, after which a further 339 records were excluded. Of the remaining 26 articles, 13 met the inclusion criteria for the final analysis.^14–26^ The study selection process is outlined in Figure 1. Among the 13 studies, 12 reported on colorectal cancer surgery while one, by Mäkelä-Kaikkonen *et al*, reported on rectal prolapse surgery.^20^ As this is an operation that does not involve colectomy, it was not included in the meta-analysis of the remaining 12 studies but its findings are summarised together with the other 12 studies in Table 2.

**Table 2:** Summary of the 13 included trials and their reported outcomes

Study	Country	Total patients	Surgery	Outcome	Robotic events	Robotic patients	Laparoscopic events	Laparoscopic patients
Baik, 2008^14^	South Korea	36	Rectal cancer	Postoperative complications	4	18	1	16
				Operative time (mins)	Mean 217.1 SD 51.6	18	Mean 204.3 SD 51.9	16
				LOS (days)	Mean 6.9 SD 1.3	18	Mean 8.7 SD 1.3	16
				Blood loss (ΔHg g/dl)	Mean 0.6 SD 0.6	18	Mean 0.8 SD 1.0	16
				Conversions	0	18	2	16
				LN retrieved	Mean 20.0 SD 9.1	18	Mean 17.4 SD 10.6	16
Debakey, 2018^15^	Egypt	45	Rectal cancer	Postoperative complications	6	21	7	24
				Operative time (mins)	Median 201 Range 140–280	21	Median 134.5 Range 110–190	24
				LOS (days)	Median 3 Range 2–14	21	Median 2 Range 2–11	24
				Blood loss (ml)	Median 200 Range 50–650	21	Median 325 Range 100–800	24
				Conversions	1	21	2	24
				Positive CRM	18	21	15	24
				LN retrieved	Median 14 Range 8–20	21	Median 13 Range 9–21	24
Feng, 2022^16^	China	1,171	Rectal cancer	Postoperative complications	95	586	135	585
				Operative time (mins)	Median 173 IQR 140–225	586	Median 170 IQR 140–209	585
				LOS (days)	Median 7 IQR 7–11	586	Median 8 IQR 7–12	585
				Blood loss (ml)	Median 40 IQR 30–100	586	Median 50 IQR 40–100	585
				Conversions	10	586	23	585
				Positive CRM	22	547	39	543
Jayne, 2017^17^	International	471	Rectal cancer	Postoperative complications	78	237	73	234
				Operative time (mins)	Mean 298.5 SD 88.71	236	Mean 261.0 SD 83.24	230
				LOS (days)	Mean 8.0 SD 5.85	237	Mean 8.2 SD 6.03	230
				Conversions	19	236	28	230
				Positive CRM	12	235	14	224
Jiménez Rodríguez, 2011^18^	Spain	56	Sigmoid and rectal cancer	Postoperative complications	4	28	4	28
				Operative time (mins)	Mean 159.4 SD 43.5	28	Mean 135.1 SD 29.2	28
				LOS (days)	Mean 9.3 SD 8.1	28	Mean 9.2 SD 6.8	28
				Conversions	2	28	2	28
				LN retrieved	Mean 17.6 SD 9.2	28	Mean 14.9 SD 8.7	28
Kim, 2018^19^	South Korea	139	Rectal cancer	Postoperative complications	23	66	17	73
				Operative time (mins)	Mean 339.2 SD 80.1	66	Mean 227.8 SD 65.6	73
				LOS (days)	Mean 10.3 SD 3.4	66	Mean 10.8 SD 7.4	73
				Blood loss (ml)	Median 100 Range 0–1,000	66	Median 50 Range 0–300	73
				Conversions	1	66	0	73
				Positive CRM	4	66	4	73
				LN retrieved	Median 18 Range 7–59	66	Median 15 Range 4–40	73
Mäkelä-Kaikkonen, 2016^20^	Finland	30	Rectal prolapse	Postoperative complications	5	16	1	14
				Operative time (minutes)	Mean 125 SD 27	16	Mean 130 SD 25	14
				LOS (days)	Mean 2.2 SD 1.5	16	Mean 2.5 SD 0.9	14
Park, 2012^21^	South Korea	70	Colonic cancer	Postoperative complications	6	35	7	35
				Operative time (mins)	Mean 195 SD 41	35	Mean 130 SD 43	35
				LOS (days)	Mean 7.9 SD 4.1	35	Mean 8.3 SD 4.2	35
				Blood loss (ml)	Mean 35.8 SD 26.3	35	Mean 56.8 SD 31.3	35
				Conversions	0	35	0	35
				LN retrieved	Mean 29.9 SD 14.7	35	Mean 30.8 SD 13.3	35
Park, 2013^22^	South Korea	80	Rectal cancer	Postoperative complications	6	40	5	40
				Operative time (mins)	Mean 235.5 SD 57.5	40	Mean 185.4 SD 72.8	40
				LOS (days)	Mean 10.6 SD 4.2	40	Mean 11.3 SD 3.6	40
				Blood loss (ml)	Mean 45.7 SD 40.0	40	Mean 59.2 SD 35.8	40
				Conversions	0	40	0	40
				Positive CRM	3	40	2	40
				LN retrieved	Mean 12.9 SD 7.5	40	Mean 13.3 SD 8.6	40
Park, 2019^23^	South Korea	71	Colonic cancer	Postoperative complications	6	35	7	36
				Operative time (mins)	Mean 195 SD 41	35	Mean 129.7 SD 43.2	36
				LOS (days)	Mean 7.9 SD 4.1	35	Mean 8.3 SD 4.2	36
				Blood loss (ml)	Mean 35.8 SD 36.3	35	Mean 46.8 SD 31.3	36
				Conversions	0	35	0	36
				LN retrieved	Mean 29.9 SD 14.7	35	Mean 30.8 SD 13.3	36
Patriti, 2009^24^	Italy	66	Rectal cancer	Postoperative complications	9	29	7	37
				Operative time (mins)	Mean 202 SD 12	29	Mean 208 SD 7	37
				LOS (days)	Mean 11.9 SD 7.5	29	Mean 9.6 SD 6.9	37
				Blood loss (ml)	Mean 137.4 SD 156	29	Mean 127 SD 169	37
				Conversions	0	29	7	37
				LN retrieved	Mean 10.3 SD 4	29	Mean 11.2 SD 5	37
Tang, 2020^25^	China	130	Rectal cancer	Postoperative complications	7	66	8	64
				Blood loss (ml)	Mean 73.4 SD 49.7	66	Mean 119.1 SD 65.7	64
				Conversions	0	66	0	64
				Positive CRM	1	66	0	64
				LN retrieved	Mean 15.7 SD 6.2	66	Mean 13.8 SD 6.1	64
Wang, 2016^26^	China	137	Rectal cancer	Postoperative complications	8	71	10	66
				Operative time (mins)	Median 246.9 Range 210–330	71	Median 207.3 Range 170–230	66
				LN retrieved	Median 16.4 Range 8–33	71	Median 16.3 Range 7–39	66
CRM = circumferential resection margin; IQR = interquartile range; LN = lymph nodes; LOS = length of stay; SD = standard deviation								

### Individual study particulars

Studies spanning over 13 years (from 2008 to 2022) were reviewed. The included studies involved 2,500 patients, of whom 1,248 were in the robotic group and 1,252 were in the laparoscopic group. Encompassing diverse geographic locations, the studies originated from six countries (China, Egypt, Finland, Italy, South Korea and Spain), with one international study involving ten countries.

### Risk of bias assessment

**Figure 2:**
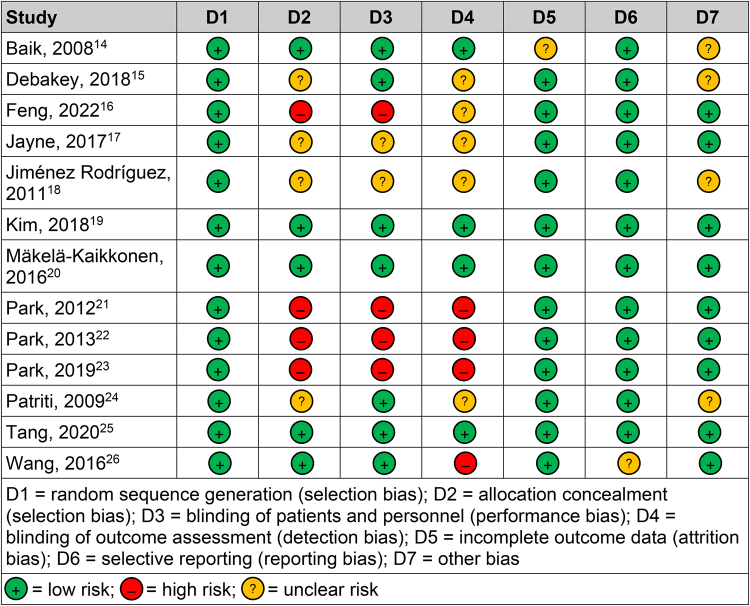
Cochrane risk of bias assessment^13^

The results of the risk of bias assessments are summarised in Figure 2. All of the 13 included studies had a low risk of bias for random sequence generation, with patients being assigned randomly to the two groups. Four studies were categorised as being at high risk of selection bias as there was no allocation concealment while in four studies, there was an unclear risk.

In four studies, both patients and personnel were unblinded, resulting in a high risk of performance bias. In two studies, an unclear risk of performance bias was noted owing to insufficient information about the blinding process. Regarding the blinding of outcome assessment, five studies had an unclear risk of detection bias, with four studies having a high risk due to lack of blinding.

Attrition bias was noted to be of low risk in all the studies except one, in which the risk was unclear owing to inadequate information on outcome data. Only one study had an unclear risk of reporting bias, with all other studies having a low risk of bias for selective reporting. There was an unclear risk of other bias reported in four studies, with all others having a low risk.

### Primary outcome: postoperative complications

**Figure 3:**
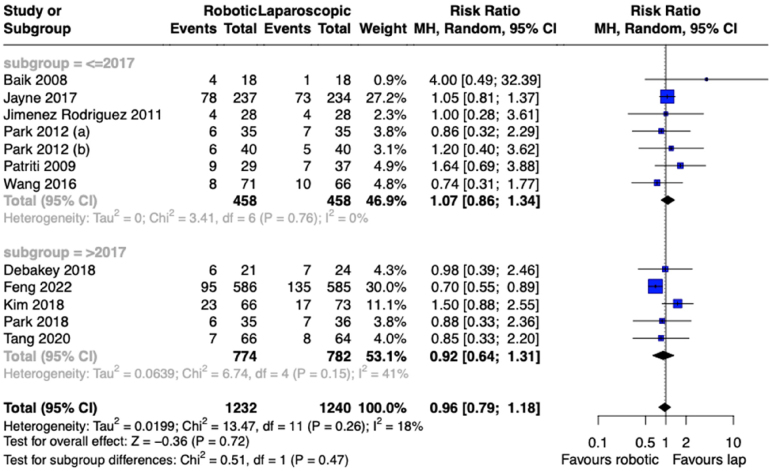
Meta-analysis of overall postoperative complications for included trials with subgroup analysis of reports published in 2017 or earlier and of reports published after 2017

Short-term perioperative postoperative complications were reported in all studies, resulting in an overall complication rate of 21.05% for robotic surgery and 22.73% for laparoscopic surgery. Meta-analysis revealed no significant difference between the two groups (RR: 0.96, 95% CI: 0.79 to 1.18, *p*=0.72), with low heterogeneity noted among the studies (I^2^=18%, *p*=0.26) (Figure 3).

**Figure 4:**
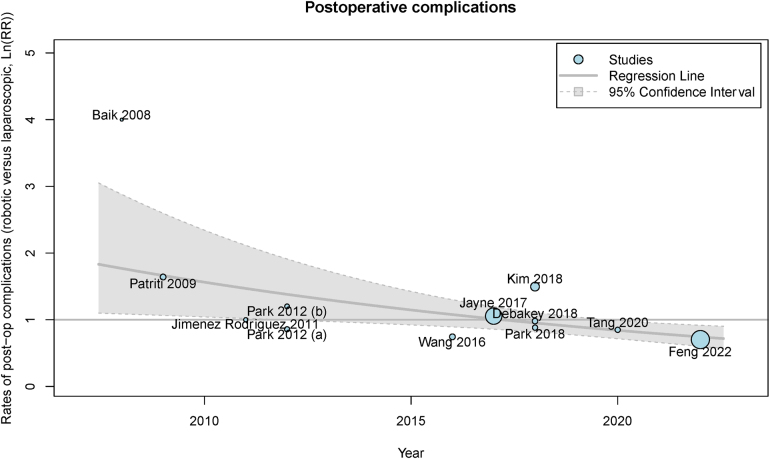
Meta-regression of overall postoperative complications for included trials

Mixed-effects meta-regression was employed to assess whether there were any temporal trends in complication rates for robotic and laparoscopic procedures. This showed that the difference in rate of postoperative complications between robotic and laparoscopic approaches changed significantly over time, in favour of the robotic group (yearly change in Ln(RR): −0.0620, 95% CI: −0.1057 to −0.0183, *p*=0.005) (Figure 4).

### Secondary outcomes

Secondary postoperative outcome measures reported in the included RCTs were operative time, blood loss, number of lymph nodes retrieved, positive circumferential resection margins, conversion to open surgery and length of hospital stay. All had common outcome metrics, except for blood loss, where both millilitres of blood loss and change in haemoglobin were used.

**Figure 5:**
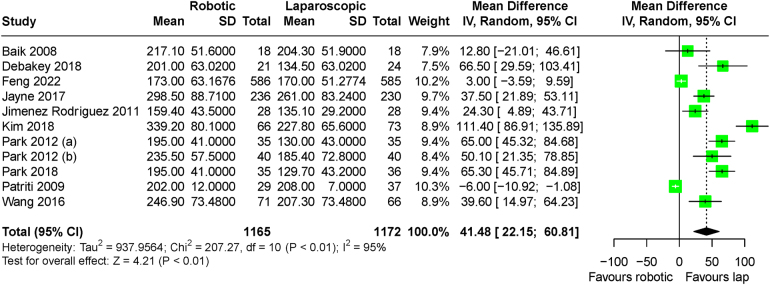
Meta-analysis of operative time for included trials

**Figure 6:**
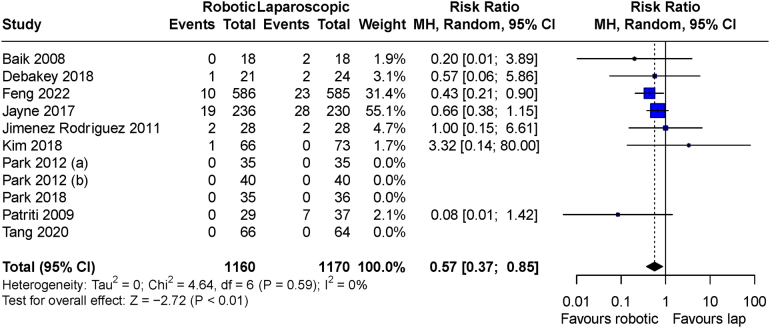
Meta-analysis of conversions for included trials

The only outcomes that demonstrated a difference in meta-analysis between the robotic and laparoscopic groups were operative time and conversion to open surgery. Operative time was significantly shorter in the laparoscopic arms (MD: 41.48 minutes, 95% CI: 22.15 to 60.81 minutes, *p*<0.001), with significant heterogeneity observed among the studies (I^2^=95%, *p*<0.001) (Figure 5). Conversions were significantly less likely to occur in the robotic arms (RR: 0.57, 95% CI: 0.37 to 0.85, *p*=0.007), with no significant heterogeneity (I^2^=0%, *p*=0.59) (Figure 6). No secondary outcomes showed a significant trend over time in meta-regression analysis. All secondary outcome analysis results are outlined in Table 3.

**Table 3:** Summary of results for secondary outcome measures with 95% confidence intervals

	Meta-analysis	*p*-value	Favours	Meta-regression	*p*-value	Favours
Operative time (MD, mins)	41.48 (22.15 to 60.81)	**<0.001**	Laparoscopic	2.57 (−3.51 to 8.65)	0.41	–
LOS (MD, days)	−0.51 (−1.13 to 0.11)	0.11	–	0.03 (−0.12 to 0.19)	0.68	–
Blood loss (SMD, ml)	−0.25 (−0.55 to 0.06)	0.11	–	0.00 (−0.08 to 0.07)	0.95	–
Conversion (RR)	0.57 (0.37 to 0.85)	**0.007**	Robotic	0.00 (−0.13 to 0.12)	0.95	–
Positive CRM (RR)	0.73 (0.43 to 1.23)	0.24	–	−0.08 (−0.27 to 0.10)	0.38	–
LN retrieved (MD, number of nodes)	0.69 (−0.19 to 1.57)	0.13	–	0.20 (−0.05 to 0.44)	0.11	–
CRM = circumferential resection margin; LN = lymph nodes; LOS = length of stay; MD = mean difference; RR = relative risk; SMD = standard mean difference						

## Discussion

The paradigm in colorectal cancer treatment has historically favoured laparoscopic surgery but a discernible shift towards robotic surgery is currently underway. On performing a meta-regression, there was a significant trend towards reduced postoperative complications in the robotic groups compared with the laparoscopic groups. With complication rates being one of the crucial endpoints of any surgery, such information is key to bringing out the real time advantages of robotic surgery.

Our finding likely represents the fact that early randomised trials compared surgeons who were expert laparoscopic surgeons and relatively novice robotic surgeons whereas more recent trials compare surgeons with expertise in both techniques. This has wider ramifications and highlights that early trials on a novel surgical technique may not reflect its true potential benefits. This could provide crucial information to enhance robotic training and early skill development in this surgical technique. In the long term, it has the potential to establish this method as not only safe but also superior to conventional laparoscopy.

The first validated robotic training programme, published in 2015, has been considered the gold standard, with different specialties replicating this to meet their training needs.^27^ The use of robotic surgery has increased substantially over recent years,^28^ and it continues to evolve and grow. Longer operative times and rising costs have limited the widespread availability of the robot but in 2022, a study by Hancock *et al* showed that for elective left-sided and rectal resections, the robot proved to be most cost effective.^29^ Numerous studies have been conducted to examine the outcomes after robotic and laparoscopic surgery, and their findings have varied. However, the more recent literature has shown that these approaches are comparable,^9^ with some studies suggesting slightly improved results when performing robotic operations.^8,19,30^

To our knowledge, this study represents the first attempt to conduct a meta-analysis of RCTs incorporating meta-regression analysis to explore the evolution of outcomes in laparoscopic and robotic surgery over time. Our inclusion criteria were specific to RCTs in order to ensure the highest level of evidence. Additionally, the criteria were not limited to cancer resections; one study looked at outcomes after benign colorectal procedures.^20^ The last systematic review to include only RCTs for colorectal resections was published in 2014 by Liao *et al* but this had a small sample size of only four studies.^7^ Other meta-analyses of RCTs have been limited to rectal cancer resections, restricting the conclusions to specific colorectal operations.^9,32^ Our study has advantages over previous reviews in that it incorporates the latest RCTs, includes an additional meta-regression analysis and encompasses all colorectal procedures (both benign and malignant).

The primary outcome measure was the overall complication rate in the immediate postoperative period. Complication rates following surgery are an important outcome to assess the safety of any intervention. Following major surgery, complication rates are dependent not only on surgical expertise but also on the method of operation, instruments used and operative time as well as other miscellaneous factors. In our study, the complication rates were lower in the robotic cohort (21.05% vs 22.73%), with no significant difference between the two groups on meta-analysis. It is difficult to compare our results directly with those of previous studies as our study included a mixed sample group of benign and malignant resections. Results from prior studies have been variable.^33–36^ However, the conclusion drawn in the majority of existing studies, in line with ours, is that robotic surgery is safe when compared with laparoscopic surgery, with an acceptable complication rate.

Owing to the variability in how outcomes were reported, it was not feasible to compare postoperative complications using a systematic approach, such as the Clavien–Dindo classification system.^31^ We had planned to group postoperative complications by severity using this system but this was not possible because of the inconsistent way in which studies reported postoperative complications. It is acknowledged that this is a limitation of the current review as it may hinder generalisability of the findings. We have compared the complications in a similar way to previous published reviews. Additionally, all reported complications are listed in Table 4.

**Table 4:** List of all reported complications among the 13 included trials

Study	Group	Postoperative complications																							
		Total	Bleeding	Pain	Scrotal swelling	Anastomotic leak	Wound related	Urine/chest/line infection	Fever	Ileus	Urinary retention	Enteritis	Deep vein thrombus	Intestinal obstruction	Dehiscence	Intra-abdominal abscess	Traumatic head injury	Inguinal hernia	Ischaemic colon	Urinary complications	Respiratory complications	GI complications	Cardiac complications	Stoma related	Other
Baik, 2008^14^	RS	**4**	1	2	1																				
	LS	**1**	1																						
Debakey, 2018^15^	RS	**6**				1	2			2			1												
	LS	**7**				1	2			3															1 (erectile dysfunction)
Feng, 2022^16^	RS	**95**	8			25	18	21		5			6										12	3	7
	LS	**135**	12			37	22	30		11			9										9	4	9
Jayne, 2017^17^	RS	**78**					21													17	4	35	3		17
	LS	**73**					19													14	6	40	6		12
Jiménez Rodríguez, 2011^18^	RS	**4**	1			1											1	1							
	LS	**4**	1										1	1	1										
Kim, 2018^19^	RS	**23**	1			8	1			6	5													3	2
	LS	**17**				5		3		9														2	1
Mäkelä- Kaikkonen, 2016^20^	RS	**5**	4	1																					
	LS	**1**							1																
Park, 2012^21^	RS	**6**	1			1		2		2															
	LS	**7**	3					2		1						1									
Park, 2013^22^	RS	**6**				3					1					1			1						
	LS	**5**				2					1					1			1						
Park, 2019^23^	RS	**6**	1			1	2			1															
	LS	**7**	3				2			1						1									
Patriti, 2009^24^	RS	**9**	1			2		1		2	1	2													
	LS	**7**	2			1		2		1	1														
Tang, 2020^25^	RS	**7**																							
	LS	**8**																							
Wang, 2016^26^	RS	**8**	1			2	1	3								1									
	LS	**10**				3	3	4																	
GI = gastrointestinal; LS = laparoscopic surgery; RS = robotic surgery																									

The analysis of operative times in our study demonstrated a significant difference between the robotic and laparoscopic groups favouring the laparoscopic approach, with significant heterogeneity among the studies. Our results are similar to those from previous meta-analyses in the existing literature for colorectal surgery,^33,34,37^ with robotic surgery having a known longer operative time. This can likely be attributed to the additional setup involved in robotic procedures. This setup includes positioning the robot and docking its arms, and it is influenced by the staff’s familiarity with the robotic equipment and the associated processes.

A study conducted by Souders *et al* in 2017 found that allocation of tasks, role definition and presence of visual cues can significantly reduce robotic operating theatre turnover times.^38^ Another systematic review from 2021 compared the learning curve of robotic surgery versus laparoscopic colorectal surgery and showed that when surgeons are inexperienced on both platforms, operative times can be faster in robotic surgery.^39^ This may be due to better initial baseline ability rather than a shorter learning curve.

Conversions to open surgery were found to be significantly less common in the robotic group than in the laparoscopic cohort, with no heterogeneity among the studies. The ROLARR (RObotic versus LAparoscopic Resection for Rectal cancer) trial was one of the largest randomised trials to have looked at conversion rates as the primary endpoint for laparoscopic and robotic surgery; the results did not show any difference.^17^ However, a 2018 meta-analysis of RCTs for rectal cancer indicated a significantly lower rate of conversion to open surgery in the robotic group.^32^ Studies in the literature have also found lower conversion rates in the robotic group for right-sided colectomies.^34,36,40,41^

Our meta-analysis did not demonstrate a significant difference in the length of hospital stay between the laparoscopic and robotic groups. The existing evidence regarding this outcome is equivocal without a clear consensus. Our results differ from some previous studies where the length of stay was found to be considerably shorter in the robotic group.^7,34,42^ Nevertheless, we note other studies that have quoted opposing results.^32,33^

The present study is limited by its short-term follow-up of perioperative complications, thereby restricting the conclusions of this review to a relatively short timeframe. Another limitation arises from the potential heterogeneity in the study populations as our analysis includes both benign and malignant colon and rectal procedures. The choice of surgical approach will depend on the surgeon’s preference and the complexity of the case. Given the current state of available evidence, this represents a methodological limitation necessary to enable this type of analysis. It should be highlighted, however, that the studies included in our analysis exhibited minimal heterogeneity for the primary outcome measure.

## Conclusions

This review establishes the safety of robotic surgery, demonstrating a complication profile akin to laparoscopic surgery across the spectrum of colorectal procedures. Emerging evidence hints at a trend towards reduced postoperative complications in robotic procedures, potentially reflective of the growing expertise and mastery of the technique among surgeons. The ongoing expansion of training programmes and enhanced accessibility to robotic platforms mean we are poised to exploit the advantages of robotic surgery. Increasing uptake of robotic surgery and new evidence will further clarify its clinical efficacy, evolving the landscape of colorectal surgery.
